# *Salvadora persica* Fruit Methanolic Extract, Nutritional Profiling, Therapeutic Potential in High‐Fat Diet‐Induced Hyperlipidemic Rats: In Vivo, In Vitro, and In Silico Approaches

**DOI:** 10.1002/fsn3.71595

**Published:** 2026-03-05

**Authors:** Nudrat Khursheed, Mostafa A. Abdel‐Maksoud, Salman Alrokayan, Khurram Afzal, Muhammad Naeem Zubairi, Muhammad Tauseef Sultan, Asad Abbas, Hassan Raza, Ahmad Mujtaba Noman, Heba A. S. El‐Nashar, Mohammad S. Mubarak

**Affiliations:** ^1^ Department of Human Nutrition, Faculty of Food Science and Nutrition Bahauddin Zakariya University Multan Pakistan; ^2^ Research Chair of Biomedical applications of nanomaterials, Biochemistry Department, College of Science King Saud University Riyadh Saudi Arabia; ^3^ Department of Food Science and Technology, Faculty of Food Science and Nutrition Bahauddin Zakariya University Multan Pakistan; ^4^ Department of Pharmacognosy, Faculty of Pharmacy Ain Shams University Cairo Egypt; ^5^ Department of Chemistry The University of Jordan Amman Jordan; ^6^ Department of Chemistry Indiana University Bloomington Indiana USA

**Keywords:** antioxidant activity, fruit powder, high‐fat diet, hyperlipidemia, liver function, methanolic extract, *S. persica* fruit, serum lipid profile

## Abstract

This manuscript presents original research on *Salvadora persica* fruit, investigating its antioxidant and metabolic properties using in vivo, in vitro, and in silico approaches in high‐fat‐diet induced hyperlipidemic rat models. Nutritional profiling, phytochemical analysis (TPC, DPPH, FRAP, ABTS), and GC–MS analysis identified key bioactive compounds. The antioxidant tests revealed high activity of the methanolic extract, with TPC (62.1 mg GAE/g), DPPH (67.8%), FRAP (335.4 μmol Fe^2+^/g), and ABTS (540.2 μmol Trolox/g) assays. GC–MS analysis revealed oleic acid as the predominant compound (56.64%), followed by (9E,11E)‐octadecadienoic acid (18.10%) and *n*‐hexadecanoic acid (10.92%). Molecular docking studies confirmed strong binding affinities with HMG‐CoA reductase. Furthermore, in vivo studies in male Wistar albino rats (*n* = 5 per group) confirmed that *S. persica* significantly restored antioxidant enzyme activities (SOD, Catalase, and GSH) and reduced oxidative stress markers (MDA and NO) in a dose‐dependent manner (*p* < 0.05). Lipid parameters (TG, TC, LDL, and VLDL) in animals treated with *S. persica* were significantly reduced, and liver and kidney markers, including AST, ALT, creatinine, and urea, were significantly improved (*p* < 0.05). Additionally, glucose levels in hyperlipidemic rats treated with *S. persica* methanolic extract were lower than those in the negative control (*p* < 0.05). These results underscore the promising antioxidant, hypoglycemic, hypolipidemic, hepatoprotective, and renoprotective potential of the methanolic extract of *S. persica* .

## Graphical abstract

*Salvadora persica* methanolic extract exhibited strong antioxidant, hypolipidemic, and hypoglycemic effects in high‐fat diet‐induced hyperlipidemic rats. In vitro antioxidant assays, GC–MS profiling, and in vivo studies confirmed improvements in the lipid profile and in markers of oxidative stress, liver, and kidney function. In silico studies also confirm bioactive compounds have strong binding affinities with key lipid‐regulating targets, such as HMG‐CoA reductase.

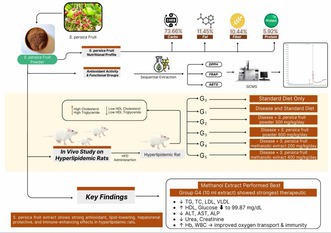

## 1 Introduction

Hyperlipidemia is a major health issue, particularly among young adults worldwide, characterized by abnormal elevations in blood lipids, particularly triglycerides and cholesterol (Aslam et al. 2022; Yanai et al. 2023). In contrast, high triglyceride levels contribute to the development of atherosclerosis, which increases the risk of cardiovascular diseases and affects life expectancy. The rising prevalence of lipid disorders is largely attributable to poor dietary habits and physical inactivity, particularly in developing countries (Giles 2024; Shen et al. 2024). As traditional lipid‐lowering medications often come with side effects, there is an increasing shift toward exploring natural alternatives that are both safe and effective for managing hyperlipidemia (Khalid et al. 2025).

Medicinal plants are widely used in traditional medicine owing to their bioactive compounds and health‐promoting mechanisms (Davis and Choisy 2024). In this regard, plant‐based interventions have garnered significant attention due to their safety, affordability, and minimal side effects compared to synthetic drugs (Shaito et al. 2020). Their therapeutic efficacy is attributed to bioactive compounds, including flavonoids, phenolic compounds, terpenoids, and essential fatty acids. These compounds are known for their antioxidant, anti‐inflammatory, and lipid‐lowering properties, which help reduce the burden of noncommunicable diseases (Mivefroshan and Afsargharehbagh 2024; Ansari et al. 2020).

*S. persica* fruit (common name: pilu, arak) is an underutilized species of the *Salvadoraceae* family, and its multipurpose uses are evidenced from folk literature and the Holy Quran (Khafagi et al. 2006). It is an ancient indigenous plant found in Yemen, Iran, Jordan, Saudi Arabia, India, and Pakistan. *S. persica* is rich in phytochemicals, antioxidants, polyphenols, and essential fatty acids and possesses hypolipidemic, anti‐inflammatory, anti‐plague, antimicrobial, analgesic, and anti‐antipyretic activities as reported in previous studies (Gul et al. 2025; Mohany et al. 2023; Shahzad et al. 2022). Moreover, recent studies reported numerous phenolic acids (gallic acid, cinnamic acid, hydroxy benzoic acid, and chlorogenic acid) and flavonoids (rutin, catechin, and myrecetin) that can potentially reduce cholesterol and combat free radicals, hence reduce oxidative damage and plaque formation (Khanam et al. 2022; Jasim and Habeeb 2025).

There is increasing demand to explore more underutilized plant species with strong traditional and medicinal uses for treating diseases such as diabetes, obesity, cancer, and cardiovascular diseases. To our knowledge, limited studies have been published that compare the antioxidant, anti‐inflammatory, and anti‐hyperlipidemic insights through high‐fat diet‐induced hyperlipidemia. In this context, the current study, for the first time, explores the methanolic extract of *S. persica* fruit and its therapeutic efficacy through in vivo, in vitro, and in silico validation. Therefore, the current study was designed to analyze the nutritional composition and antioxidant profile (DPPH, FRAP, TPC, and ABTS) of this extract. Furthermore, the phytochemical profile was quantified through gas chromatography–mass spectrometry, and in vivo and in vitro potential were also analyzed in Sprague Dawley rats in a high‐fat diet‐induced hyperlipidemia. Moreover, in silico validation was assessed by molecular docking against HMG‐CoA reductase. This multidisciplinary approach aims to establish scientific evidence for the nutritional and therapeutic efficacy of *S. persica* fruit extract in regulating lipid levels.

## 2 Materials and Methods

### 2.1 Plant Material

*S. persica* fruit was procured from Pansar market, Multan, and sent to the Food Analysis Lab, Faculty of Food Science and Nutrition of Bahauddin Zakariya University, Multan. *S. persica* was washed, dried, and ground into a fine powder using a grinder (Model BJ‐9176). The powder was then stored in polyethylene bags for further use.

### 2.2 Proximate Composition of *S. persica* Fruit

The proximate composition of *S. persica* fruit was determined according to the standard AOAC procedures (AOAC 2012). Moisture content was measured by oven drying (AOAC 925.10), and ash content by incineration in a muffle furnace (AOAC 923.03). The Kjeldahl method (AOAC 979.09) was used to estimate crude protein, and crude fat was determined by Soxhlet extraction (AOAC 920.39) using a Soxhlet apparatus (Model: NZF‐063, China). The oil fiber content was evaluated using the acid–base digestion method (AOAC 962.09), and the nitrogen‐free extract (NFE) was determined by differences.

$$\%\mathrm{NFE}=100–\left(\%\text{Protein}+\%\text{Fiber}+\%\mathrm{Ash}+\%\mathrm{Fat}+\%\text{Moisture}\right)$$

### 2.3 Antioxidant Analysis of *S. persica* Fruit

The sequential extraction of *S. persica* fruit was performed to isolate a wide range of bioactive compounds based on polarity, following the procedure outlined by Yassen et al. (2025), with slight modifications. Methanol was selected as the extraction solvent due to its high efficiency in extracting bioactive compounds, particularly phenolics and flavonoids, which are known to possess antioxidant properties (Ramadan and Alshamrani 2025).

#### 2.3.1 Total Phenolic Content (TPC)

The TPC of *S. persica* was determined by mixing 6 mL of Folin–Ciocalteau reagent with 1 mL of 4% sodium carbonate solution and 0.5 mL of each extract. The solution was kept in the dark at room temperature for 90 min, and absorbance was measured at 765 nm using a spectrophotometer. TPC was expressed in mg of gallic acid equivalents (GAE) per 100 mg of extract, as mentioned by Saeedi Borujeni et al. (2019).

#### 2.3.2 Determination of the Antioxidant Activity of *S. persica* Fruit Using the 1,1‐Diphenyl‐2‐Picrylhydrazyl (DPPH) Assay

The DPPH% inhibition of *S. persica* fruit was measured according to published procedures, with minor modifications (Ryntathiang et al. 2025). A 0.1 mM ethanol solution of DPPH was prepared, and 2 mL of extract was combined with 2 mL of DPPH solution, and the mixture was kept in the dark at room temperature. Vitamin C served as the positive control, while the negative control consisted of 2.5 mL of methanol and 1 mL of DPPH solution. Absorbance was measured at 518 nm using a spectrophotometer (Model 823‐0210 P‐2‐R). The antioxidant activity was calculated using the following formula:

$$\text{Inhibition}\;\left(\%\right)=\frac{\text{Control absorbance}-\text{Sample absorbance}}{\text{Control absorbance}}\times 100$$

#### 2.3.3 Determination of Ferric Reducing Antioxidant Power (FRAP) of *S. persica* Fruit

The antioxidant activity of *S. persica* fruit was measured using the FRAP assay, following the method of Khanam et al. (2022) with slight modifications. FRAP reagent was added with 2,4,6‐(2‐tripyridyl)‐s‐triazine (TPTZ) (10 mM), FeCl_3_ (0.02 M), and sodium acetate buffer (pH 3.7) in the ratio of 10:1:1. Then, 2 mL of FRAP reagent was mixed with 1 mL of the concentrated extract. The mixture was allowed to stand in the dark at room temperature for 30 min, with ferrous sulfate used as the standard. Absorbance was measured at 593 nm using a spectrophotometer (Model 823‐0210 P‐2‐R), and the standard curve was generated using ferrous sulfate; results were expressed in μmol of Trolox equivalent to 100 mL of sample.

#### 2.3.4 The 2,2′‐Azinobis(3‐Ethylbenzothiazoline‐6‐Sulphonate) (ABTS) Assay of *S. persica* Fruit

The radical‐scavenging activity of the *S. persica* fruit extract was determined using the 2,2′‐azinobis (3‐ethylbenzothiazoline‐6‐sulphonate) (ABTS) method, following published procedures with slight modifications (Ryntathiang et al. 2025). We prepared an ABTS+ solution by combining ABTS with potassium persulfate and allowing it to stand in the dark for 16 h. The absorbance was measured at 734 nm using a Spectrophotometer (Model 823‐0210 P‐2‐R) after incubation of a mixture of 3 mL ABTS solution and 30 μL fruit extract in the dark for 16 h at 26°C, and expressed as % inhibition (Ryntathiang et al. 2025).

### 2.4 Gas Chromatography–Mass Spectrometry (GC–MS) Analysis

The methanolic extract of *S. persica* fruit was subjected to phytochemical profiling using GC–MS analysis following the method of Yassen et al. (2025) with modifications. The analysis was conducted using a probe‐type ultrasonicator (Model VCX130, Sonics & Materials Inc., Newtown, USA), operating at 20 kHz and a power density of 98.0 W/cm^2^. The oven temperature was programmed from 60°C (held for 2 min) to 300°C at 10°C/min, with a final hold time of 10 min. High‐purity helium was used as the carrier gas at a constant flow rate of 1.0 mL/min. A 1 μL sample was injected in splitless mode. The constituents were identified by comparing their mass spectra with the NIST 14 mass spectral library.

### 2.5 Experimental Design and Animal Housing

The therapeutic potential of *S. persica* fruit in managing hyperlipidemia was evaluated using male albino rats weighing approximately 180 g. The animals were acclimated under standard laboratory conditions (25°C ± 2°C, 50% ± 10% humidity, and a 12‐h light/dark cycle) for 1 week. During this period, the rats were fed poultry feed type No. 13 and had free access to water. Physical and serological tests were performed prior to the start of the experiment (Yang et al. 2022). Experiments with animals were conducted in accordance with accepted animal husbandry standards and ARRIVE protocols, and the principles stated in the Declaration of Helsinki. Ethical approval was obtained from the Ethical Committee of the Department of Human Nutrition, Bahauddin Zakariya University, Multan (Protocol Number: REC‐029). The animals were handled humanely, ensuring their welfare throughout the research process.

### 2.6 Induction of Hyperlipidemia

Hyperlipidemia was induced in all groups except the negative control by administering a 1% cholic acid solution and coconut oil (1%) following the procedure outlined by Wang et al. (2022). After the induction period, the respective interventions, powder and extract, were initiated and administered daily for 28 days duration. Rats were monitored for food intake, weight gain, and behavior during the trial.

### 2.7 Experimental Groups and Dosing

A total of 30 rats were randomly divided into six groups of five animals each. Group 0 served as the standard‐diet control group without disease induction or intervention. Group 1 served as the negative control and was fed a standard diet without disease induction or treatment. Groups 2 and 3 were induced with hyperlipidemia using a high‐fat diet (HFD) and supplemented with *S. persica* fruit powder of 300 mg/kg/day and 600 mg/kg/day, respectively, mixed with the standard basal diet. Groups 4 and 5 were also induced with hyperlipidemia via HFD but were treated with *S. persica* fruit's methanolic extract at doses of 200 mg/kg/day and 400 mg/kg/day through oral gavage for 4 weeks to evaluate the therapeutic effects of *S. persica* on diet‐induced hyperlipidemia, as mentioned in Table 1. The dosages of *S. persica* extract and powder were selected based on body weight, prior studies, and expected therapeutic effects. The doses were normalized to mg/kg body weight for consistency and comparability with other studies in the field. The high and low doses were selected to evaluate both the therapeutic efficacy and the dose–response relationship. The methanolic extract of *S. persica* was standardized for total phenolic content prior to use in the in vivo trial (Saeedi Borujeni et al. 2019).

**TABLE 1:** Treatment groups.

Groups	Treatments
Group 1	G0: Standard diet only
Group 2	G1: Disease and standard diet
Group 3	G2: Disease + *S. persica* fruit powder 300 mg/kg bw
Group 4	G3: Disease + *S. persica* fruit powder 600 mg/kg bw
Group 5	G4: Disease + *S. persica* fruit methanolic extract 400 mg/kg bw
Group 6	G5: Disease + *S. persica* fruit methanolic extract 300 mg/kg bw

#### 2.7.1 Preparation of Standard Diet

The rats were fed a standard basal diet (recipe listed in Table 2). Treatment groups received the standard diet supplemented with *S. persica* fruit powder (300 g/Kg and 12 g/100 g), and the methanolic extract was administered by oral gavage.

**TABLE 2:** Recipe for standard diet and high‐fat diet.

Ingredients	Standard diet
Corn starch	65
Wheat bran	5
Cellulose	5
Casein protein	10
Corn oil	10
Coconut oil	—
Vitamins	2.5
Minerals	1.5
Cholic acid	—

### 2.8 Feed Intake and Body Weight

The feed intake (g) and water intake (mL) of all animals in different groups were recorded on the 7th, 14th, 21st, and 28th days. The water and feed consumption of each animal was measured by using the following formula:

$$\text{Feed consumption}\;\mathrm{per}\;\text{week}=\frac{\text{food provided}\;\left(g\right)–\text{leftover}\;\left(g\right)}{\text{number of rats in each group}}$$

$$\text{Water intake}\;\mathrm{per}\;\text{week}=\frac{\text{water provided}\;\left(\mathrm{mL}\right)–\text{leftover}\;\left(\mathrm{mL}\right)}{\text{number of rats in each group}}$$

### 2.9 Slaughtering of Animals

The rats were anesthetized through intraperitoneal injection of Ketamine hydrochloride (70 mg/kg B.W) and sacrificed through dissection after 28 days. Blood samples were collected via cardiac puncture using sterile syringes. For serum preparation, blood was transferred into serum collection tubes, allowed to clot at room temperature, and then centrifuged to obtain serum for biochemical analysis. Serum was isolated by centrifugation (3000 rpm for 5 min) and stored at −20°C in Eppendorf tubes for subsequent biochemical analysis. Major organs, including the liver, heart, kidneys, lungs, and spleen, were carefully dissected, cleaned, and weighed using a precision balance (GX‐600, Japan) following the method of Khan et al. (2014).

### 2.10 Blood Analysis

#### 2.10.1 Serum Biochemistry and Hematological Profile

For hematological evaluation, blood was collected into EDTA‐coated vials, where EDTA acted as an anticoagulant to prevent clotting, and whole blood samples were used for hematological profiling. The blood was aspirated into the Cobas 6000 Auto analyzer for quantification of red blood cell (RBC) count (×10^6^/μL), hemoglobin (Hb) in g/dL, hematocrit (%), white blood cell (WBC) count (×10^3^/μL), and differential WBC counts (Khanam et al. 2022). For serum biochemistry, blood was drawn into gel vials without anticoagulants. The serum and plasma were subsequently separated by centrifuging the clotted blood at 3500 rpm for 10 min using a centrifuge (HERMLE Labortechnik GmbH, D‐78564, Z 326 K, Germany). Plasma was used to measure liver function tests, including alanine aminotransferase (ALT) and aspartate aminotransferase (AST), alkaline phosphatase (ALP), and bilirubin, and kidney function tests (Urea and Creatinine), using the Lisa 300 Hycel automaton (Austria) (Yang et al. 2022).

#### 2.10.2 Serum Lipid and Protein Profile

The blood lipid profile was assessed by measuring triglycerides (TG) in mg/dL, total cholesterol (TC) in mg/dL, high‐density lipoprotein (HDL) in mg/dL, low‐density lipoprotein (LDL) in mg/dL, and very low‐density lipoprotein (VLDL) in mg/dL. On the other hand, serum protein levels, including albumin and globulin (g/dL), were measured using an automated chemistry analyzer (BS‐240 VET, Mindray, China), as described by Saeedi Borujeni et al. (2019).

### 2.11 Determination of Oxidative Stress Markers

#### 2.11.1 Preparation of Tissue Homogenates

The rat liver tissues of all treatment groups (weighing 0.25 g) were homogenized with phosphate‐buffered saline (PBS) solution (1 mL of 50 mM) with 0.1 M EDTA through centrifugation for 20 min at 4°C at 12,000 × g. Subsequently, the supernatant was separated, and tissue homogenates were used to determine protein concentration using bovine serum albumin (standard) according to the Bradford (1976) method.

#### 2.11.2 Determination of Malondialdehyde (MDA)

MDA level in liver tissue homogenates (homogenized in 0.9% NaCl) was quantified using the thiobarbituric acid reactive substances (TBARS) method as outlined by Buege and Aust (1978).

#### 2.11.3 Determination of Nitric Oxide (NO)

NO levels in liver tissues were determined using the Griess reaction, as described by Yilmaz et al. (2005), with minor modifications. Liver homogenates were mixed with sulfanilic acid and naphthyl ethylenediamine to form a pink‐colored product. The absorbance of the resulting solution was measured at 540 nm, and NO levels were expressed as μmol/L.

#### 2.11.4 Glutathione (GSH)

The reduced glutathione assay was performed by mixing 62.5 μL liver homogenate, 187.5 μL phosphate buffer (0.2 M and pH 8.2), and 12.5 μL dithiobis‐nitrobenzoic acid (0.01 M), followed by 987.5 μL methanol. The mixture was shaken at 240 rpm for 15 min using a laboratory mixer, then centrifuged at 300 rpm for 15 min, and the absorbance was measured at 412 nm. GSH level was expressed as mM GSH/Lg tissue as described by Sedlak and Lindsay (1968).

#### 2.11.5 Catalase Activity (CAT)

The CAT activity was evaluated by monitoring the decomposition of hydrogen peroxide. Briefly, 1 μL of rat liver homogenate was diluted in 0.1 M PBS (pH 7), and a 2 mM hydrogen peroxide solution was added to reach a final volume of 1 mL in the cuvette. Absorbance changes were measured at 240 nm over 2 min at 30‐s intervals, with CAT activity calculated using the molar coefficient (*ɛ* = 43.6 Mcm^−1^) and expressed as U/mg protein (Raza et al. 2025).

#### 2.11.6 Superoxide Dismutase (SOD)

SOD activity was measured by assessing its ability to inhibit the photochemical reduction of nitrotetrazolium chloride (NBT). Superoxide anion, generated through riboflavin‐mediated illumination, reacts with SOD, reducing formazan product formation. The assay mixture consisted of 10 μL of liver tissue homogenate, 641 μL of PBS (0.067 M) at pH 7.0, 40 μL EDTA (0.1 M), 20 μL of NBT (1.5 mM), and 9 μL of 0.1 mM riboflavin. The mixture was illuminated with a 40‐watt lamp for 15 min, and absorbance was measured at 560 nm using a spectrophotometer. One SOD unit is defined as the amount of enzyme required to inhibit 50% of NBT reduction, expressed as U/mg protein (Bouhalit and Kechrid 2018).

$$\text{Inhibition}\;\left(\%\right)=\frac{\text{Absorbance of control}–\text{absorbance of sample}}{\text{Control}\;}\times 100$$

#### 2.11.7 Cardiac Troponin‐I

Cardiac Troponin‐I, a specific marker of myocardial injury, was quantified using a commercially available rat‐specific ELISA kit (Feldman and Liang 2019).

### 2.12 In Silico Validation

#### 2.12.1 Protein and Ligand Preparation

The crystal structure of human HMG‐CoA reductase (PDB ID: 1HW8) at 2.10 Å resolution was obtained from the Protein Data Bank (PDB) and prepared by removing nonessential molecules and adding hydrogens. Water molecules beyond 5 Å from the co‐crystal ligand were deleted, while catalytic waters were retained in a parallel set to assess water‐mediated interactions. Ligands (gallic acid, rutin, and isolinoleic acid (9E,11E)‐octadecadienoic acid) with the following PubChem CID 370, 5,280,805, and 5,282,796 were downloaded from PubChem in 3D SDF format. The ligands were then converted to 3D using Open Babel, minimized with the MMFF94 force field, and saved as PDBQT files with defined rotatable bonds.

#### 2.12.2 Molecular Docking Studies

To elucidate the potential molecular mechanism of action, molecular docking was performed using a protocol similar to that of Khalid et al. (2025). The 3D structures of the predominant bioactive compounds (gallic acid, rutin, and (9E,11E)‐octadecadienoic acid) and a positive control (atorvastatin) were obtained from PubChem using the following PubChem IDs: 370, 5,280,805, 5,282,796, and 60,823, respectively, and were optimized using the Avogadro software. The crystal structures of the key therapeutic target, HMG‐CoA reductase (PDB ID: 1HW8), were obtained from the RCSB Protein Data Bank. The proteins were prepared by removing water molecules and adding polar hydrogens. Docking simulations were performed with AutoDock Vina, and the binding interactions were visualized in PyMOL.

### 2.13 Statistical Analysis

All experiments were performed in triplicate, and data were expressed as the mean ± standard deviation (SD) as described by Yassen et al. (2025). The statistical analysis was performed using Statistix 10 (Analytical Software, USA), and one‐way ANOVA was used to compare the therapeutic potential of *S. persica* (fruit powder and methanolic extract) for hyperlipidemia, oxidative stress markers, serum proteins, and liver function. A completely randomized design (CRD) followed by a Tukey HSD post hoc test for mean comparisons was used to assess significance at the 5% level.

## 3 Results

### 3.1 Proximate Composition (100 g, dry Weight Basis)

The nutritional composition of *S. persica* fruit, including its high carbohydrate and moderate fat content, is detailed in Table 3. Results showed that *S. persica* fruit has a high carbohydrate content (73.66%), along with crude fat (11.45%) and crude fiber content (10.44%). It also contains ash (9.37%), crude protein (5.92%), and moisture content (8.12%). These results highlight its potential as a nutrient‐dense and energy‐rich plant source.

**TABLE 3:** Proximate analysis of *S. persica* fruit.

Nutritional composition	Mean ± SD
Moisture (%)	8.12 ± 0.35
Crude protein (%)	5.92 ± 0.21
Crude fat (%)	11.45 ± 0.36
Crude fiber (%)	10.44 ± 0.42
Ash (%)	9.37 ± 0.31
Carbohydrates (%)	73.66 ± 1.34

### 3.2 Antioxidant Activity of *S. рersiса* Fruit

The antioxidant activity of the *S. persica* fruit extract was assessed using the TPC, DPPH, FRAP, and ABTS assays across four solvents: water, methanol, ethanol, and acetone; results are shown in Table 4. Our findings indicate that the *S. persica* fruit extract in methanol exhibits the highest antioxidant activity in DPPH (67.8%), FRAP (335.4 μmol Fe^2+^/g), TPC (62.1 mg GAE/g), and ABTS (540.2 μmol Trolox/g). Ethanol and acetone extracts also exhibited strong antioxidant potential, whereas the water extract showed the lowest activity. These results suggest that methanol is the most effective solvent for extracting antioxidant‐rich compounds from *S. persica* fruit.

**TABLE 4:** Antioxidant activity of *S. persica* fruit extracts.

Solvent	DPPH (% inhibition)	FRAP (μmol Fe^2+^/g)	TPC (mg GAE/g)	ABTS (μmol Trolox/g)
Water	42.3 ± 1.2^a^	220.5 ± 4.8^c^	29.5 ± 1.64^c^	430.7 ± 8.9^b^
Acetone	58.4 ± 1.6^c^	298.6 ± 6.2^b^	44.6 ± 3.48^b^	505.3 ± 10.4^c^
Ethanol	63.1 ± 1.4^ab^	312.7 ± 5.9^ab^	53.5 ± 4.63^a^	525.1 ± 9.7^a^
Methanol	67.8 ± 1.5^b^	335.4 ± 7.1^a^	62.1 ± 2.85^a^	540.2 ± 11.2^a^

*Note*: Means having similar superscripts in columns do not differ significantly.

### 3.3 Identification of Phytochemicals by GC–MS

**FIGURE 1:**
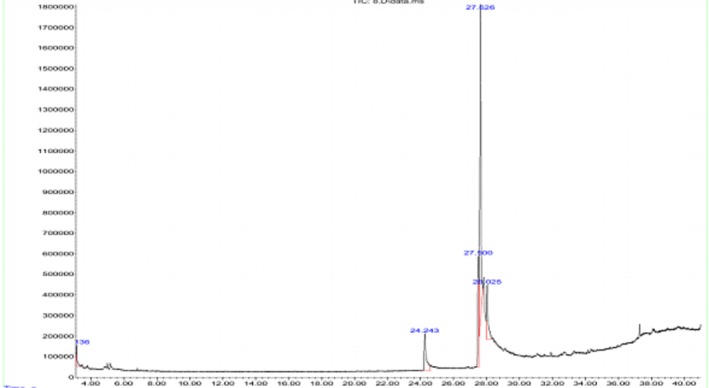
GC–MS profiling of bioactive compounds.

GC–MS analysis identified various bioactive compounds in the methanolic extract of *S. persica* , as shown in Figure 1. Our results indicated that the methanolic extract contains oleic acid as the predominant compound, accounting for 56.64%, followed by (9E,11E)‐octadecadienoic acid (18.10%) and *n*‐hexadecanoic acid (10.92%). Other compounds detected included hydrazine (2.43%), 2‐methyl‐2‐pentanol (2.43%), and 3‐methyl‐6‐ethyl‐2,4‐dioxadecane (2.43%), all present at lower concentrations as mentioned in Table 5.

**TABLE 5:** GC–MS profiling of bioactive compounds.

No.	Compound	Formula	Retention time (min)	Molecular weight (g/mol)	% Area
1	Hydrazine, 1,1‐dimethyl‐2‐(1‐methylbutyl)	C_6_H_14_N_2_	3.136	112.0835	2.43
2	2‐Pentanol, 2‐methyl	C_5_H_12_O	3.136	90.1462	2.43
3	3‐Methyl‐6‐ethyl‐2,4‐dioxadecane	C_16_H_30_O_2_	3.136	258.4	2.43
4	*n*‐Hexadecanoic acid	C1_6_H_32_O_2_	24.243	256.4	10.92
5	(9E,11E)‐Octadecadienoic acid	C_18_H_32_O_2_	27.500	280.4	18.10
6	Oleic Acid	C_18_H_34_O_2_	27.626	282.5	56.64
7	10E,12Z‐Octadecadienoic acid	C_18_H_32_O_2_	28.025	280.4	11.91

### 3.4 Feeԁ and Water Intаke

**FIGURE 2:**
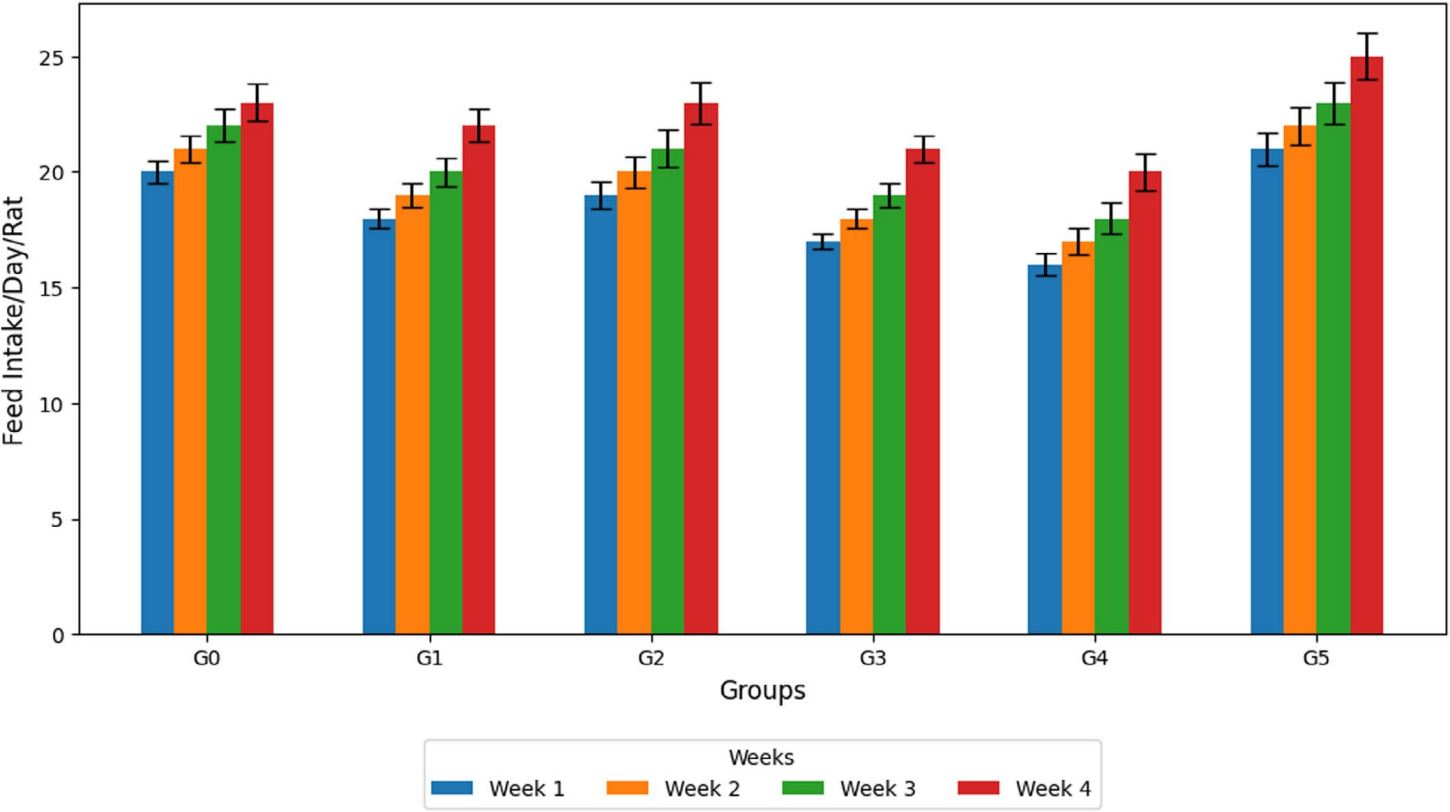
Feed (g) intake/day/rat.

**FIGURE 3:**
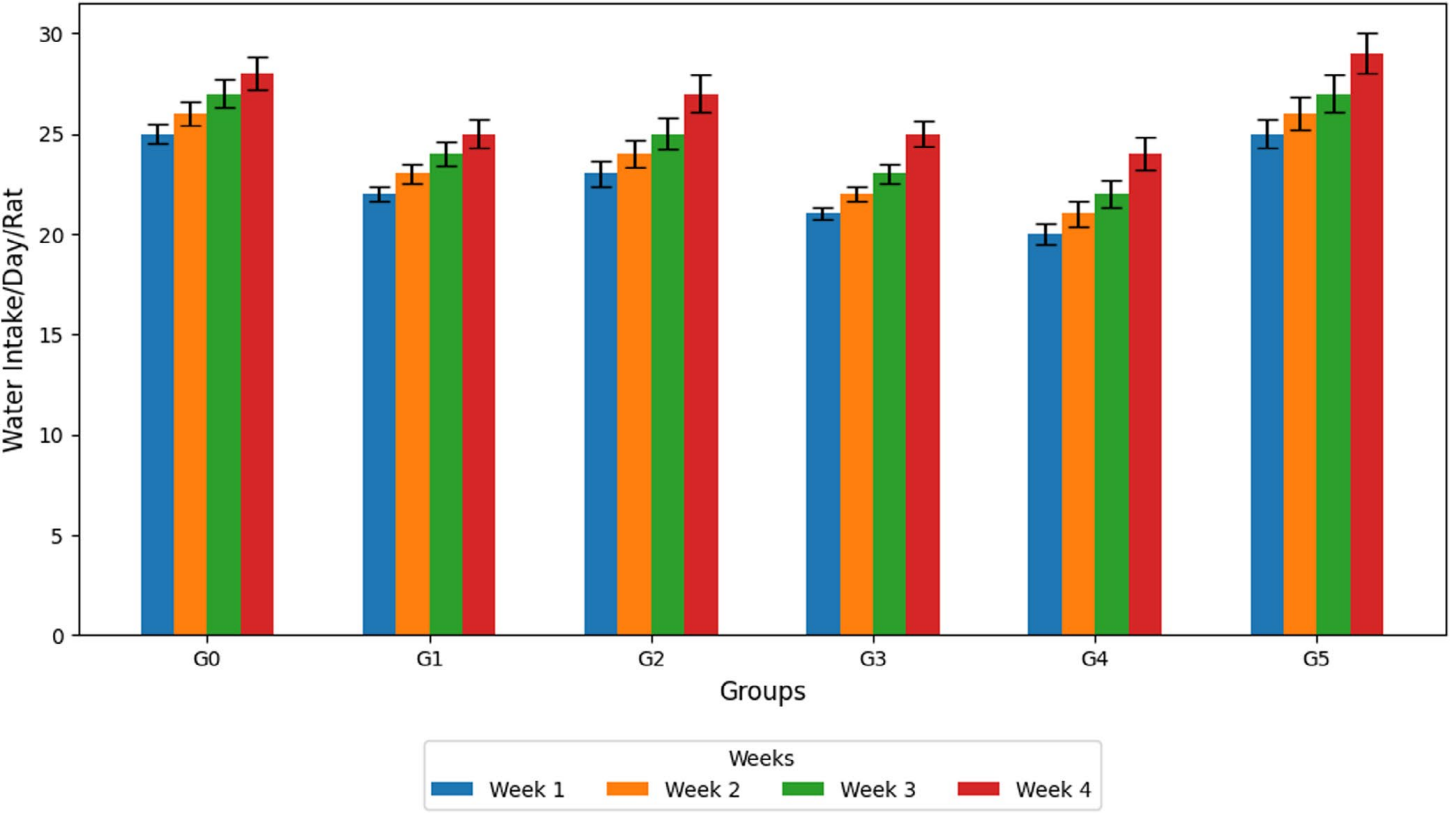
Water (mL) intake/day/rat.

**FIGURE 4:**
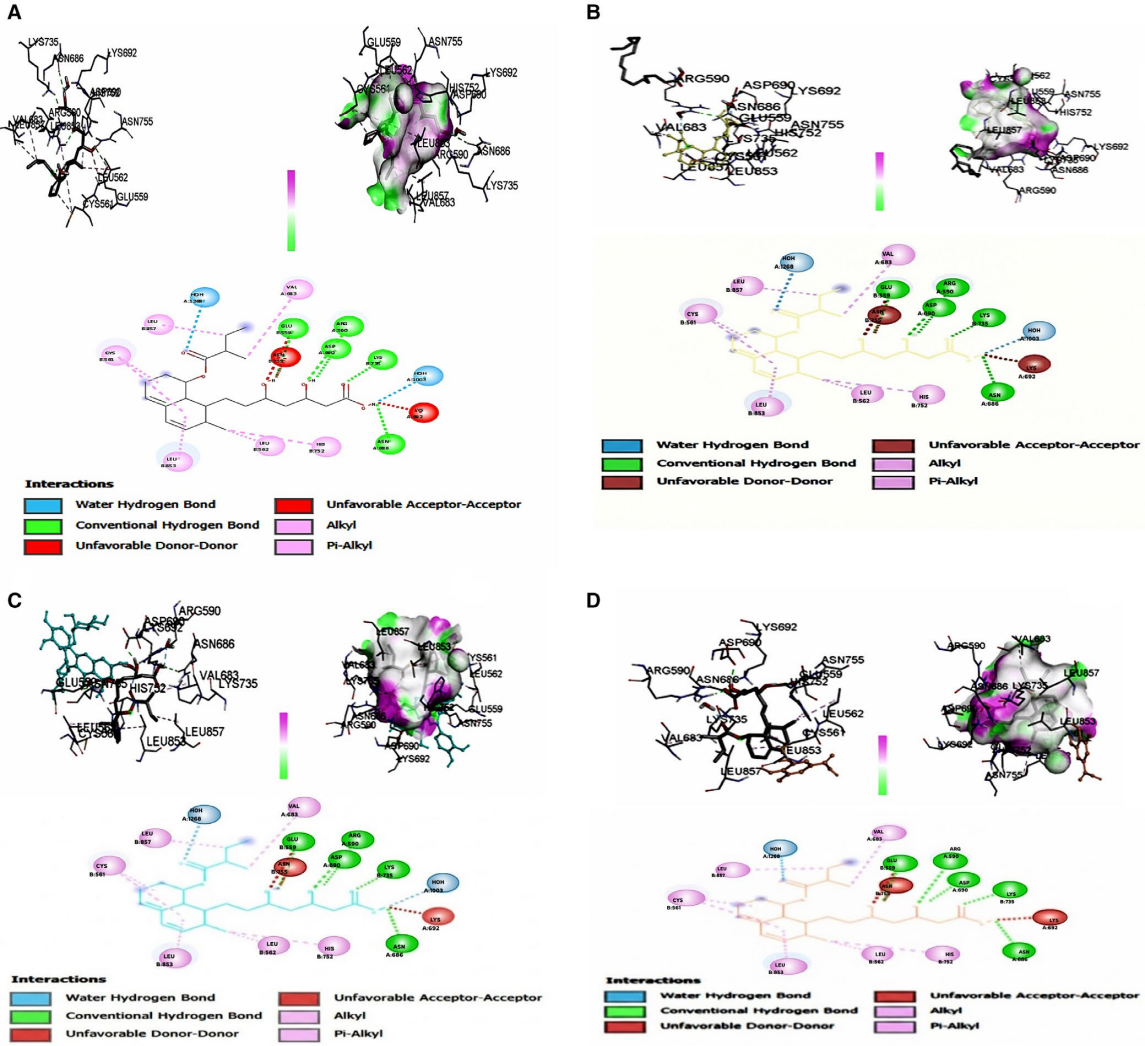
(A) Molecular docking insights of Atorvastatin and HMG‐CoA reductase. The structures are shown in ligand–protein interaction, hydrogen bond interactions, and 2D structure, respectively. (B) Molecular docking insights of (9E,11E)‐octadecadienoic acid and HMG‐CoA reductase. The structures are shown in ligand–protein interaction, hydrogen bond interactions, and 2D structure, respectively. (C) Molecular docking insights of rutin and HMG‐CoA reductase. The structures are shown in ligand–protein interaction, hydrogen bond interactions, and 2D structure, respectively. (D) Molecular docking insights of gallic acid and HMG‐CoA reductase. The structures are shown in ligand–protein interaction, hydrogen bond interactions, and 2D structure, respectively.

The mean comparisons of feed and water intake across treatment groups and treatment durations are presented in Figures 2 and 3. The G5 group fed 300 mL/kg of the methanolic extract of *S. persica* showed significant differences compared with the control group over 4 weeks (*p* < 0.05). Moreover, disease groups showed a slight decrease in feed intake, with G3 exhibiting the lowest intake throughout the study period, perhaps due to increased dietary fiber, which may have reduced satiety. The hyperlipidemic rats fed the methanolic extracts (G4 and G5) exhibited a moderate intake, indicating good palatability and tolerance. Likewise, water consumption was highest in G0, and the disease‐control groups showed slightly lower values. Nonetheless, the G4 and G5 treatment groups showed slow progress in water intake at Week 4, indicating that their metabolic balance was partially restored (Figures 4A–D).

### 3.5 Body and Organ Weights

The effect of *S. persica* fruit powder and extract treatments on body weight and relative organ weights of the rats is presented in Table 6. Results revealed that in the HFD group (G1), body weight increased significantly compared with the normal control group (G0) (*p* < 0.01). Treatment with *S. persica* extract resulted in a significant, dose‐dependent reduction in body weight, with the high‐dose extract group (G5) weighing 171.44 ± 7.47 g, the closest to G0 (*p* < 0.05). Similarly, the liver of rats in the HFD group exhibited hepatomegaly, with a liver weight of 8.19 ± 0.49 g, whereas the methanolic extract of *S. persica* reduced liver weight, with the G5 group showing a liver weight of 7.26 ± 0.57 g.

**TABLE 6:** Effect of *S. persica* fruit and extract intake on body and organ weight in rats.

Parameters	Body weight (g)	Kidney R (g)	Kidney L (g)	Liver (g)	Heart (g)	Lungs (g)	Spleen (g)
G0	155.88 ± 2.70^d^	0.44 ± 0.02^a^	0.35 ± 0.02^b^	7.11 ± 0.19^b^	0.43 ± 0.03^d^	1.18 ± 0.04^a^	0.43 ± 0.02^a^
G1	260.71 ± 9.03^a^	0.48 ± 0.01^a^	0.40 ± 0.01^a^	8.19 ± 0.49^a^	0.51 ± 0.00 ^a^	1.14 ± 0.06^a^	0.48 ± 0.01^a^
G2	229.91 ± 17.96^ab^	0.49 ± 0.00 ^a^	0.39 ± 0.01^ab^	7.74 ± 0.27^ab^	0.50 ± 0.02^ab^	1.19 ± 0.05^a^	0.47 ± 0.02^a^
G3	185.11 ± 10.31^cd^	0.46 ± 0.02 ^a^	0.37 ± 0.01^ab^	7.41 ± 0.37^ab^	0.46 ± 0.01 ^bcd^	1.16 ± 0.01^a^	0.45 ± 0.01^a^
G4	202.21 ± 14.15^bc^	0.47 ± 0.04 ^a^	0.38 ± 0.01^ab^	7.59 ± 0.27^ab^	0.48 ± 0.00^abc^	1.17 ± 0.06^a^	0.46 ± 0.04 ^a^
G5	171.44 ± 7.47^cd^	0.45 ± 0.02^a^	0.36 ± 0.01^b^	7.26 ± 0.57^ab^	0.45 ± 0.01^cd^	1.15 ± 0.05^a^	0.44 ± 0.02^a^
*F* ratio	35.19 **	2.25 ns	4.50*	3.07 ns	11.05*	0.41 ns	2.10 ns

*Note:* *, significant; **, highly significant; Means having similar superscripts in columns do not differ significantly.

Abbreviations: G0, standard diet only; G1, disease and standard diet; G2, disease + *S. persica* fruit powder (300 mg/kg bw); G3, disease + *S. persica* fruit powder (600 mg/kg bw); G4, disease + *S. persica* fruit methanolic extract (200 mg/kg bw); G5, disease + *S. persica* fruit methanolic extract (400 mg/kg/day); ns, nonsignificant.

### 3.6 Serum Glucose and Lipid Profile

The comparative effects of *S. persica* fruit powder and methanolic extract showed significant reductions in blood glucose and lipid parameters (TG, TC, HDL, LDL, and VLDL), as shown in Table 7. In contrast, the treatment groups showed a significant reduction in serum glucose, VLDL, and triglycerides, while no significant change was observed in serum HDL and LDL. The high‐dose group fed on 400 mL/kg methanolic extract of *S. persica* (G5) exhibited significantly (*p* < 0.05) lower levels of serum glucose (102.35 ± 6.71 mg/dL), TG (91.54 ± 0.00 mg/dL), TC (94.69 ± 4.31 mg/dL), and LDL (37.41 ± 2.08 mg/dL) compared to the HFD group (G1). Moreover, HDL levels in the G5 group increased to 38.97 ± 1.56 mg/dL.

**TABLE 7:** Effect of *S. persica* fruit powder and extract intake on glucose and lipid profile in rats.

Parameters	Glucose (mg/dL)	TG (mg/dL)	TC (mg/dL)	HDL (mg/dL)	LDL (mg/dL)	VLDL (mg/dL)
G0	88.66 ± 2.66^e^	77.21 ± 1.54^e^	90.26 ± 1.81^c^	39.21 ± 1.96^a^	35.61 ± 1.88 c	15.44 ± 0.94^d^
G1	165.41 ± 7.58^a^	141.33 ± 6.16^a^	109.87 ± 6.68^a^	35.87 ± 1.86^a^	45.73 ± 2.86 a	28.27 ± 0.75^a^
G2	141.67 ± 8.85^b^	126.43 ± 2.19^b^	104.82 ± 4.80^ab^	37.77 ± 1.36^a^	41.77 ± 0.72^ab^	25.28 ± 1.41^ab^
G3	129.45 ± 4.48^bc^	117.89 ± 6.13^bc^	102.79 ± 3.71^abc^	38.23 ± 0.00^a^	40.98 ± 1.79^abc^	23.58 ± 1.70^bc^
G4	117.43 ± 7.33^cd^	109.34 ± 6.56^c^	100.04 ± 7.94^abc^	38.61 ± 1.68^a^	39.56 ± 3.09^bc^	21.87 ± 1.00^c^
G5	102.35 ± 6.71^de^	91.54 ± 0.00^d^	94.69 ± 4.31^bc^	38.97 ± 1.56^a^	37.41 ± 2.08^bc^	18.31 ± 0.48^d^
*F* ratio	52.51**	78.12**	5.47*	1.83^ns^	7.73^ns^	51.99**

*Note:* *, significant; **, highly significant. Means having similar superscripts in columns do not differ significantly.

Abbreviations: G0, standard diet only; G1, disease and standard diet; G2, disease + *S. persica* fruit powder (300 mg/kg bw); G3, disease + *S. persica* fruit powder (600 mg/kg bw); G4, disease + *S. persica* fruit methanolic extract (200 mg/kg bw); G5, disease + *S. persica* fruit methanolic extract (400 mg/kg/day); ns, nonsignificant.

### 3.7 Oxidative Stress and Cardiac Markers

The restoration of antioxidant enzymes (SOD, GSH, Catalase) and reduction in oxidative stress markers across different treatment groups are depicted in Table 8. SOD activity was significantly higher in the G5 group (7.91 ± 0.29 U/mL) than in the G1 (disease control) group (4.77 ± 0.27 U/mL), indicating compromised antioxidant defense. Our findings showed that antioxidant defense progressively improves with increasing doses of *S. persica* extract. Similarly, catalase activity increased in all treatment groups, with the highest activity in G5 (16.66 ± 0.88 U/mL), suggesting strengthened antioxidant defense. In addition, MDA levels were lower across all treatment groups compared with the control group (G1), with the G5 group (2.67 ± 0.03 nmol/mL) showing the most pronounced reduction, indicating a protective effect against oxidative damage. GSH was also notably elevated in G5 (3.91 ± 0.18 nmol/mg), indicating enhanced detoxification of reactive oxygen species and reduced oxidative stress. In contrast, NO levels were reduced across all treatment groups, with the G5 group showing a significant reduction (31.66 ± 1.58 μmol/L), followed by G4, G3, and G2. Moreover, Troponin I improved across all treatment groups, particularly in the G5 group (0.07 ± 0.01 ng/mL), suggesting potential cardioprotective benefits.

**TABLE 8:** Effect of *S. persica* fruit powder and extract on oxidative stress and cardiac markers in rats.

Parameters	SOD (U/mL)	Catalase (U/mL)	MDA (nmol/mL)	GSH (nmol/mg)	NO (μmol/L)	Troponin I (ng/mL)
G0	8.65 ± 0.69^a^	17.78 ± 0.36^a^	2.39 ± 0.06^e^	4.21 ± 0.26^a^	25.89 ± 1.29^e^	0.05 ± 0.01^e^
G1	4.77 ± 0.27^d^	9.45 ± 0.66^c^	4.56 ± 0.05^a^	2.34 ± 0.13^d^	54.78 ± 2.19^a^	0.18 ± 0.01^a^
G2	6.19 ± 0.28^c^	14.98 ± 0.15^b^	3.78 ± 0.23^b^	2.78 ± 0.22^cd^	49.11 ± 2.60^ab^	0.14 ± 0.01^b^
G3	7.11 ± 0.26^bc^	16.05 ± 1.00^ab^	2.91 ± 0.25^cd^	3.33 ± 0.20^b^	38.56 ± 2.53^c^	0.09 ± 0.01^cd^
G4	6.88 ± 0.12^c^	15.56 ± 0.62^b^	3.29 ± 0.03^c^	3.09 ± 0.05^bc^	44.71 ± 1.95^b^	0.11 ± 0.01^c^
G5	7.91 ± 0.29^ab^	16.66 ± 0.88^ab^	2.67 ± 0.03^de^	3.91 ± 0.18^a^	31.66 ± 1.58^d^	0.07 ± 0.01^de^

*Note:* Means having similar superscripts in columns do not differ significantly.

Abbreviations: G0, standard diet only; G1, disease and standard diet; G2, disease + *S. persica* fruit powder (300 mg/kg bw); G3, disease + *S. persica* fruit powder (600 mg/kg bw); G4, disease + *S. persica* fruit methanolic extract (200 mg/kg bw); G5, disease + *S. persica* fruit methanolic extract (400 mg/kg/day); ns, nonsignificant.

### 3.8 Liver Functions

The liver functioning test (Bilirubin, AST, ALT, and ALP) indicates hepatoprotective effects of *S. persica* fruit powder and extracts, as shown in Table 9. Our results from this investigation showed that in the HFD group (G1), liver enzymes were elevated, particularly AST (119.78 ± 4.79 U/L), ALT (68.66 ± 2.06 U/L), and bilirubin (0.61 ± 0.02 g/L). In contrast, treatment with *S. persica* extract (400 mL/kg) resulted in significant reductions (*p* < 0.01) in AST (71.23 ± 1.23 U/L), ALT (44.78 ± 3.88 U/L), and bilirubin (0.29 ± 0.00 g/L) compared with other groups.

**TABLE 9:** Effect of *S. persica* fruit powder and extract on liver function.

Parameters	Bilirubin (g/L)	AST (U/L)	ALT (U/L)	ALP (IU/L)
G0	0.23 ± 0.01^e^	61.19 ± 2.80^d^	39.16 ± 0.39^e^	133.67 ± 4.01^e^
G1	0.61 ± 0.02^a^	119.78 ± 4.79^a^	68.66 ± 2.06^a^	240.17 ± 6.35^a^
G2	0.47 ± 0.01^b^	95.18 ± 3.81^b^	61.17 ± 4.24^ab^	202.67 ± 12.16^b^
G3	0.33 ± 0.02^d^	77.44 ± 4.10^c^	49.65 ± 0.86^cd^	171.66 ± 7.87^c^
G4	0.39 ± 0.02^c^	86.98 ± 1.74^b^	55.14 ± 3.62^bc^	188.64 ± 5.66^bc^
G5	0.29 ± 0.00^d^	71.23 ± 1.23^c^	44.78 ± 3.88^de^	155.88 ± 7.14^d^
*F* ratio	258.88**	115.00**	41.24**	71.94**

*Note:* **, highly significant; Means having similar superscripts in columns do not differ significantly.

Abbreviations: G0, standard diet only; G1, disease and standard diet; G2, disease + *S. persica* fruit powder (300 mg/kg bw); G3, disease + *S. persica* fruit powder (600 mg/kg bw); G4, disease + *S. persica* fruit methanolic extract (200 mg/kg bw); G5, disease + *S. persica* fruit methanolic extract (400 mg/kg/day); ns, nonsignificant.

### 3.9 Serum Protein Levels

Results of total protein, albumin, globulin analysis, and their ratio in the serum of experimental rats are presented in Table 10. Results indicated no significant effect of *S. persica* fruit powder and extract on total protein, albumin, and globulin in the treatment groups. The G1 group had a protein level of 6.85 ± 0.25 g/dL, and the G5 group had 6.94 ± 0.36 g/dL. On the other hand, the A/G ratio was also significantly different, with the HFD group (G1) showing a significant decrease (1.08 ± 0.03) compared with the normal control group (G0: 1.24 ± 0.06). The other treatment groups (G2–G5), by contrast, showed a slight improvement in the A/G ratio, particularly the high‐dose group (G5), which had an A/G ratio of 1.20 ± 0.09.

**TABLE 10:** Effect of *S. persica* fruit powder and extract intake on total protein in rats.

Parameters	Total protein (g/dL)	Albumin (g/dL)	Globulin (g/dL)	A/G ratio
G0	7.03 ± 0.31^a^	3.89 ± 0.21^a^	3.14 ± 0.06 ^a^	1.24 ± 0.06^a^
G1	6.83 ± 0.25^a^	3.54 ± 0.14^a^	3.29 ± 0.03^a^	1.08 ± 0.03^b^
G2	6.86 ± 0.00^a^	3.61 ± 0.31^a^	3.25 ± 0.15^a^	1.11 ± 0.04^ab^
G3	6.89 ± 0.54^a^	3.67 ± 0.17^a^	3.22 ± 0.15^a^	1.14 ± 0.04^ab^
G4	6.90 ± 0.32^a^	3.71 ± 0.06^a^	3.19 ± 0.06^a^	1.17 ± 0.04^ab^
G5	6.94 ± 0.36^a^	3.79 ± 0.33^a^	3.15 ± 0.11^a^	1.20 ± 0.09^ab^
*F* ratio	0.13^ns^	0.93^ns^	1.00 ^ns^	3.56*

*Note:* *, significant; Means having similar superscripts in columns do not differ significantly.

Abbreviations: G0, standard diet only; G1, disease and standard diet; G2, disease + *S. persica* fruit powder (300 mg/kg bw); G3, disease + *S. persica* fruit powder (600 mg/kg bw); G4, disease + *S. persica* fruit methanolic extract (200 mg/kg bw); G5, disease + *S. persica* fruit methanolic extract (400 mg/kg/day); ns, nonsignificant.

### 3.10 Effects on Hematological Parameters

Results related to the effect of *S. persica* methanolic extract on red blood cells and white blood cell differential count are presented in Tables 11 and 12, respectively. The hematological analysis revealed that intake of the *S. persica* methanolic extract significantly affected platelet count, hematocrit (HCT), and mean corpuscular volume (MCV). There was a significant increase in platelet count in the G1 group (6.83 ± 0.18 × 10^3^/μL) and a decrease in platelet count in the G5 group (5.44 ± 0.05 × 10^3^/μL). However, there were no significant changes in RBC count or hemoglobin level across all groups, with the G5 group showing slightly higher RBC count (7.65 ± 0.35 × 10^3^/μL) and hemoglobin level (13.39 ± 0.48 g/dL). Additionally, the WBC count levels were also higher in the G1 group (8.65 ± 0.40 × 10^3^/μL), which is an indication of inflammation caused by hyperlipidemia. *S. persica* extract treatment of the G3 and G5 groups decreased the number of WBC (G5: 7.55 ± 0.35 × 10^3^/μL), which indicates a possible anti‐inflammatory effect. These findings indicate that *S. persica* supplementation may improve overall blood health by normalizing platelet count and reducing inflammation.

**TABLE 11:** Effect of *S. persica* fruit powder and extract intake on the hematological analysis in rats.

Parameter	RBC count (*10^6^ /μL)	Hb (g/dL)	Platelets count	HCT (%)	MCV (fL)	MCH (pg)	MCHC (g/dL)
G0	7.79 ± 0.13^a^	13.56 ± 0.36^a^	5.21 ± 0.18^e^	41.43 ± 1.44^a^	53.18 ± 1.06^a^	17.40 ± 0.61^a^	32.73 ± 1.18^a^
G1	6.92 ± 0.37^a^	12.19 ± 0.61^a^	6.83 ± 0.18^a^	39.78 ± 1.73^a^	57.49 ± 1.15^a^	17.61 ± 0.88^a^	30.64 ± 1.40^a^
G2	7.17 ± 0.19^a^	12.72 ± 0.25^a^	6.31 ± 0.28^b^	40.51 ± 2.03^a^	56.50 ± 1.49^a^	17.74 ± 0.45^a^	31.40 ± 2.20^a^
G3	7.46 ± 0.65^a^	13.05 ± 0.65^a^	5.94 ± 0.06^bc^	40.89 ± 0.82^a^	54.81 ± 2.51^a^	17.49 ± 0.80^a^	31.92 ± 3.32^a^
G4	7.34 ± 0.22^a^	12.91 ± 0.72^a^	5.71 ± 0.10^cd^	41.11 ± 1.42^a^	56.01 ± 1.68^a^	17.59 ± 0.30^a^	31.40 ± 0.83^a^
G5	7.65 ± 0.35^a^	13.39 ± 0.48^a^	5.44 ± 0.05^de^	41.27 ± 1.09^a^	53.95 ± 2.47^a^	17.50 ± 1.09^a^	32.43 ± 1.72^a^
*F* ratio	2.33^ns^	2.50^ns^	39.86**	0.51^ns^	2.40^ns^	0.07 ^ns^	0.46^ns^

*Note:* **, highly significant; Means having similar superscripts in columns do not differ significantly.

Abbreviations: G0, standard diet only; G1, disease and standard diet; G2, disease + *S. persica* fruit powder (300 mg/kg bw); G3, disease + *S. persica* fruit powder (600 mg/kg bw); G4, disease + *S. persica* fruit methanolic extract (200 mg/kg bw); G5, disease + *S. persica* fruit methanolic extract (400 mg/kg/day); ns, nonsignificant.

**TABLE 12:** Effect of *S. persica* fruit and extract intake on white blood cells in rats.

Parameters	WBC count (10^3^/μL)	Neutrophils (%)	Lymphocytes (%)	Monocytes (%)	Eosinophils (%)	Basophils (%)
G0	7.33 ± 0.15^b^	39.67 ± 1.59^a^	56.52 ± 3.15^a^	2.45 ± 0.17^d^	1.25 ± 0.05^a^	0.11 ± 0.00^e^
G1	8.65 ± 0.40^a^	35.33 ± 2.47^a^	58.56 ± 2.03^a^	4.56 ± 0.21^a^	1.39 ± 0.04^a^	0.16 ± 0.00^a^
G2	8.19 ± 0.30^ab^	37.11 ± 1.70^a^	57.67 ± 3.05 ^a^	3.71 ± 0.29 ^b^	1.36 ± 0.05^a^	0.15 ± 0.00^ab^
G3	7.91 ± 0.29^ab^	37.77 ± 1.36 ^a^	57.39 ± 4.14 ^a^	3.37 ± 0.15^bc^	1.33 ± 0.08 ^a^	0.14 ± 0.01^bc^
G4	7.77 ± 0.61 ^ab^	39.09 ± 1.35^a^	56.38 ± 3.52^a^	3.09 ± 0.08^c^	1.31 ± 0.06^a^	0.13 ± 0.00^cd^
G5	7.55 ± 0.35^b^	38.11 ± 1.91^a^	57.66 ± 2.08^a^	2.84 ± 0.25^cd^	1.27 ± 0.01^a^	0.12 ± 0.01^d^
*F* ratio	4.73 **	2.25^ns^	0.21^ns^	40.15**	3.15*	44.80**

*Note:* *, significant; **, highly significant; Means having similar superscripts in columns do not differ significantly.

Abbreviations: G0, standard diet only; G1, disease and standard diet; G2, disease + *S. persica* fruit powder (300 mg/kg bw); G3, disease + *S. persica* fruit powder (600 mg/kg bw); G4, disease + *S. persica* fruit methanolic extract (200 mg/kg bw); G5, disease + *S. persica* fruit methanolic extract (400 mg/kg/day); ns, nonsignificant.

### 3.11 Molecular Docking Analysis

The binding interactions and energies of key bioactive compounds (isolinoleic acid, rutin, gallic acid) with the target protein are summarized in Table 13 and depicted in Figure 4. Molecular docking was performed to evaluate interactions between the major bioactive compounds from *S. persica* and key lipid‐metabolism targets. The results indicated that rutin exhibits a high binding energy of −9.7 kcal/mol. In contrast, the binding energies of gallic acid and atorvastatin were −6.2 and −2.1 kcal/mol, respectively, indicating a strong affinity for HMG‐CoA reductase, a vital enzyme in cholesterol metabolism. In contrast, isolinoleic acid exhibited the lowest binding energy of −4.6 kcal/mol, indicating that it formed conventional hydrogen bonds, alkyl, and pi‐alkyl bonds with the key amino acid residues, Lys735, CYS561, ARG590, LEU853, VAL683, HIS752, and Glu559, which indicated that they inhibit cholesterol synthesis.

**TABLE 13:** Molecular docking and bonding specifications of bioactive compounds.

Compound	Amino acid	Distance	Category	Types	Energy
Isolinoleic acid	CYS561	5.10168	Hydrophobic	Alkyl	−4.6 kcal/mol
	LYS735	1.65757	Hydrogen bond	Conventional hydrogen bond	
	ARG590	2.37267	Hydrogen bond	Conventional hydrogen bond	
	LEU853	4.74469	Hydrophobic	Alkyl	
Rutin	HOH1268	2.60514	Hydrogen bond	Water hydrogen bond; conventional hydrogen bond	−9.7 kcal/mol
	ARG590	2.37267	Hydrogen bond	Conventional hydrogen bond	
	LYS735	1.65757	Hydrogen bond	Conventional hydrogen bond	
	HIS752	4.86611	Hydrophobic	Pi‐Alkyl	
Gallic acid	ARG590	2.37267	Hydrogen bond	Conventional hydrogen bond	−6.2 kcal/mol
	LYS735	1.65757	Hydrogen bond	Conventional hydrogen bond	
	CYS561	4.25613	Hydrophobic	Alkyl	
	VAL683	4.27201	Hydrophobic	Alkyl	
Atorvastatin	ARG59	2.37267	Hydrogen bond	Conventional hydrogen bond	−6.2 kcal/mol
	LYS735	1.65757	Hydrogen bond	Conventional hydrogen bond	
	ASN68	2.62203	Hydrogen bond	Conventional hydrogen bond	
	GLU5	3.0411	Hydrogen bond	Conventional hydrogen bond	

## 4 Discussion

Hyperlipidemia is a major contributor to the development of cardiovascular diseases and can be managed through natural treatments, owing to phytochemicals that possess lipid‐lowering and antioxidant properties. The underutilization of *S. persica* fruit in the treatment of hyperlipidemia represents a missed opportunity to leverage nature's therapeutic potential. With growing concerns over the side effects of synthetic medications, the exploration of natural treatments offers a promising, safer alternative for managing hyperlipidemia effectively. Our findings indicate that *S. persica* fruit powder is a potential energy‐dense dietary supplement with fiber and carbohydrate content. These findings are consistent with Khanam et al. (2022) and Kumari and Parida (2016), who highlighted *S. persica* fruit carbohydrate content as a natural energy source. Furthermore, the moderate protein content (5.29%) is consistent with a previous study by Hooda et al. (2014), which emphasized the importance of plant‐based protein in regulating metabolic function.

Results of the current study showed that *S. persica* fruit exhibits significant antioxidant potential in methanol among the solvents tested in TPC, DPPH, FRAP, and ABTS assays. The methanol extract exhibited the highest antioxidant activity, with 67.8% inhibition in the DPPH radical scavenging assay, whereas the FRAP and TPC assays yielded values of 335.4 μmol Fe^2+^/g and 62.1 mg GAE/g, respectively. These findings corroborate the antioxidant activity previously observed in the methanolic extracts of *S. persica* by Kumari et al. (2017) and Khanam et al. (2022). The presence of bioactive compounds, such as phenolics and flavonoids, enhances the capacity of *S. persica* fruit to scavenge free radicals and mitigate oxidative stress, which is a key factor in the development of cardiovascular disease and metabolic disorders (Rauf et al. 2022; Azemi et al. 2022; Alrasheedi and Hijazi 2020). Notably, the potent antioxidant properties of *S. persica* fruit may confer significant protection against oxidative damage, further supporting its therapeutic potential.

Furthermore, oleic acid, identified as the most abundant bioactive compound in the *S. persica* extract (56.64%), plays a significant role in modulating lipid metabolism. In this context, oleic acid may function as a bioactive component of the extract, distinct from its role as a common dietary fatty acid. This distinction clarifies its role in lipid‐regulatory therapeutic outcomes, which is inconsistent with previous research (Saeedi Borujeni et al. 2019). The specific role of oleic acid as a modulator of lipid metabolism further reinforces the therapeutic potential of *S. persica* in metabolic disorders.

In vivo studies demonstrated significant changes in water and feed intake within treatment groups. Neither the control nor the high‐dose group (G5) exhibited significant fluctuations in feed intake, indicating that the observed effects on lipid metabolism and oxidative stress markers were not attributable to reduced food and water intake. However, the HFD group showed a significant increase in body weight, indicating that the high‐fat diet induced obesity. In contrast, *S. persica* treatment reduced body weight, particularly in the high‐dose groups, suggesting that it controlled body weight by regulating fat metabolism. The liver weight, which was higher in the HFD group, also decreased in the treatment groups, further supporting the hepatoprotective effect of *S. persica* (Giles 2024).

In this study, supplementation with the methanolic extract of *S. persica* resulted in significant improvements in lipid profiles and liver function. The methanol extract treatment (G5) significantly decreased TG, TC, and LDL levels and elevated HDL levels. These findings are consistent with those of Hooda et al. (2014) and Saeedi Borujeni et al. (2019), who reported that root and *S. persica* fruit extracts exhibit hypolipidemic potential in rodent models. In addition, the decreased glucose level in the G5 group indicates a hypoglycaemic effect, suggesting that the therapy can be effective for treating diabetic dyslipidemia (Khan et al. 2014; Kumari and Parida 2016).

The hepatoprotective effects of *S. persica* were evidenced by lower levels of liver enzymes (AST, ALT, and ALP) and bilirubin in the treatment groups. These biomarkers are key to assessing liver health, and their decline suggests that *S. persica* may protect against diet‐induced liver damage (Bokhary et al. 2022; Ayoub et al. 2021). The G5 group had the lowest enzyme concentrations: AST 71.23 ± 1.23 U/L, ALT 44.78 ± 3.88 U/L, and bilirubin 0.29 ± 0.00 g/L, compared with the normal control group. The results support observations of the hepatoprotective effect of *S. persica* , indicating that it can reduce liver damage and enhance liver function and recovery in individuals with hyperlipidemia (Saeedi Borujeni et al. 2019; Elhassaneen et al. 2024; Ibrahim et al. 2011). These findings provide strong evidence of *S. persica* hepatoprotective properties, highlighting its potential for use in managing liver‐related diseases.

Hematological analysis showed that *S. persica* intake significantly influenced blood parameters. The elevated platelet count in the HFD group was normalized in the treatment groups, particularly in G5, suggesting that *S. persica* may modulate platelet function. The platelet normalization of this effect may also indicate *S. persica* , as elevated platelet activation is typically associated with inflammation (Alaraj et al. 2021; Wati and Deen 2018; Naseem et al. 2021). Additionally, the WBC count was significantly reduced in the treatment groups, with the most pronounced effect observed in G5, indicating that the methanolic extract reduced systemic inflammation in rodents. This diminution in WBC levels also indicates an immunomodulatory effect of *S. persica* , which may contribute to its anti‐inflammatory properties and its defense against chronic inflammation characteristic of cardiovascular diseases (Bokhary et al. 2022; Ibrahim et al. 2011; ELhabal et al. 2022).

Molecular docking studies revealed that rutin, gallic acid, and isolinoleic acid exhibited strong binding affinities for the key lipid‐regulating target, HMG‐CoA reductase. Rutin showed a binding energy of −9.7 kcal/mol with HMG‐CoA reductase, suggesting its potential to inhibit cholesterol biosynthesis. Recent in silico studies on rutin from *Hibiscus rosa‐sinensis* have demonstrated its potential to inhibit α‐glucosidase, with a binding energy of −8.2 kcal/mol, thereby supporting its potential health benefits (Raza et al. 2025). Additionally, studies on other plants, such as *Withania somnifera* , have shown that w‐04, the key bioactive compound of *W. somnifera*, exhibits DPP‐4 inhibitory activity with a binding energy of −6.4 kcal/mol (Khalid et al. 2025). Thus, these findings showed that medicinal plants and their bioactive compounds have the potential to ameliorate different disorders. Taken together, our findings indicate that rutin is a potential therapeutic agent for hyperlipidemia. However, atorvastatin and gallic acid exhibit similar binding affinities.

Despite the promising results, this study has some limitations. The experimental duration was limited to 4 weeks, which may not fully reflect the long‐term efficacy or potential adverse effects of *S. persica* supplementation. Additionally, the study was conducted exclusively on male albino Wistar rats, which may not fully represent the human metabolic response. In addition, hydrazine was detected in the methanolic extract at a concentration of 2.43%. While hydrazine is known to be toxic, it may also be an artifact of the extraction process. This potential toxicity has not been considered via such a study. Future studies should extend the experimental duration and include both male and female animals to assess gender‐based differences in metabolic response. Moreover, histopathological examination of vital organs, such as the liver and kidneys, is needed to validate physiological recovery at the tissue level. Additionally, isolating and quantifying individual phytochemicals responsible for the observed therapeutic effects would provide more insight into the precise mechanisms of action.

## 5 Conclusions

This study demonstrates that the methanolic fruit extract of *S. persica* exhibits significant antioxidant potential and therapeutic efficacy against hyperlipidemia. Both powder and extract forms showed dose‐dependent improvements in lipid profiles, inflammatory markers, cardiac enzymes, liver enzymes, kidney markers, and total protein levels, with the highest dose (400 mL/kg/day) approaching normal control values. Notably, *S. persica* extract significantly reduced triglycerides, total cholesterol, LDL cholesterol, and glucose levels, while increasing HDL cholesterol. In silico studies also confirm that bioactive compounds have strong binding affinities with key lipid‐regulating targets, such as HMG‐CoA reductase. It also demonstrated hepatoprotective effects by reducing liver enzyme levels and bilirubin. The fruit extract exhibited potent hypoglycemic, hypolipidemic, hepatoprotective, and reno‐protective effects. These findings support the use of *S. persica* fruit extract as a functional dietary component or adjunct herbal therapy for the management of hyperlipidemia and associated metabolic disorders. Further research, including clinical trials, is needed to confirm its therapeutic potential.

## Author Contributions

Conceptualization: N.K., K.A., and A.A.; methodology: N.K., M.N.Z. (Muhammad Naeem Zubairi), M.T.S., K.A., and A.A. (Asad Abbas); validation: M.A.A.‐M., S.A., M.N.Z., H.A.S.E.‐N., and M.S.M.; formal analysis: M.A.A.‐M., S.A., H.A.S.E.‐N., and M.S.M.; data curation: M.A.A.‐M., S.A., and H.A.S.E.‐N.; writing – original draft: K.A., M.T.S., and A.A.; writing – review and editing: N.K., K.A., and M.S.M. All authors have read and agreed to the published version of the manuscript.

## Funding

Ongoing Research Funding Program‐Research Chairs (ORF‐RC‐2026‐2600), King Saud University, Riyadh, Saudi Arabia.

## Ethics Statement

Experiments with animals of the current research were performed in accordance with Internationally Accepted Guidelines for Animal Research as prescribed by the Declaration of Helsinki. Ethical approval was obtained from the Research Ethics Committee of the Department of Human Nutrition, Bahauddin Zakariya University, Multan, Pakistan (Approval Date: 12th April 2024; Protocol Number: REC‐029).

## Conflicts of Interest

The authors declare no conflicts of interest.

## Data Availability

Data will be available on request from the corresponding author.
